# Association of serum iron status with MASLD and liver fibrosis

**DOI:** 10.1371/journal.pone.0319057

**Published:** 2025-04-01

**Authors:** Wenying Guo, Ting Weng, Yufei Song

**Affiliations:** 1 Ningbo medical center Lihuili Hospital of Ningbo University, Ningbo, Zhejiang, People’s Republic of China; Universita degli Studi della Campania Luigi Vanvitelli Scuola di Medicina e Chirurgia, Italy

## Abstract

**Background:**

The MASLD proposal updates and supplements the previous definition of NAFLD, making it more suitable for addressing the current understanding of chronic liver diseases. This study aims to investigate the potential association between serum iron status and the occurrence of MASLD and liver fibrosis.

**Methods:**

An in-depth analysis was conducted using the 2017–2020 NHANES data. To assess the relationship between serum iron status and the prevalence of MASLD and liver fibrosis, we performed comprehensive data analysis. This approach accounts for multiple variables, enhancing the robustness and reliability of our results by reducing potential confounding factors.

**Results:**

Our application of linear regression models provided significant insights through a comprehensive data analysis. Elevated serum ferritin, TIBC, and UIBC showed a distinct positive correlation with CAP, while only serum ferritin was positively correlated with LSM. Multivariate logistic regression analysis revealed that elevated levels of serum ferritin, TIBC, and UIBC were significantly associated with the occurrence of MASLD, whereas only serum ferritin showed a similar association with the occurrence of liver fibrosis.

**Conclusion:**

This study highlights the significant positive correlation between elevated levels of serum ferritin, TIBC, and UIBC with CAP and the prevalence of MASLD. A similar relationship was observed between serum ferritin with LSM and the prevalence of liver fibrosis.

## 1. Introduction

Non-alcoholic fatty liver disease (NAFLD) refers to the pathological accumulation of fat in the liver in the absence of excessive alcohol consumption [1]. This term encompasses a spectrum of liver diseases, ranging from simple hepatic steatosis to non-alcoholic steatohepatitis, which may further progress to fibrosis and cirrhosis [1]. However, NAFLD has been criticized for its lack of specificity and inability to reflect the complex etiology of the disease, posing challenges for accurate diagnosis and research [2]. Consequently, metabolic-associated fatty liver disease (MAFLD) was introduced to more accurately reflect the underlying metabolic dysfunction [2]. MAFLD emphasizes the metabolic basis of fatty liver disease, incorporating diagnostic criteria that include metabolic syndrome traits such as obesity, type 2 diabetes (T2DM), and other metabolic abnormalities. Unlike NAFLD, which is primarily defined by the exclusion of other causes of liver disease, MAFLD adopts a more inclusive approach with positive diagnostic criteria, enabling improved diagnostic precision [3]. This reclassification seeks to provide a more precise and inclusive disease definition, enhancing patient management and treatment strategies [4]. The term metabolic-associated steatotic liver disease (MASLD) has recently emerged, refining liver disease classification by focusing on metabolic factors contributing to liver steatosis [5]. MASLD streamlines diagnostic criteria, expands metabolic associations, and eliminates specific exclusions, allowing for a more inclusive diagnosis and improved disease prevention and management [5]. MASLD differs from both NAFLD and MAFLD by eliminating the exclusion of alcohol consumption and other liver diseases, allowing for a more comprehensive and flexible diagnostic framework that better aligns with clinical realities [6]. The diagnostic criteria for MASLD include the presence of hepatic steatosis, either through imaging or histology, along with at least one of the following metabolic risk factors: overweight, obesity, T2DM, dyslipidemia, or hypertension [6]. This inclusive approach facilitates early identification of individuals at risk and enables more personalized management strategies for MASLD. By simplifying diagnostic criteria and focusing on metabolic dysfunction, MASLD supports broader and more inclusive research and clinical applications [6].

Iron metabolism is a tightly regulated process that is vital for maintaining physiological balance. Iron is essential for oxygen transport, DNA synthesis, and cellular respiration, but excess iron can be toxic [7]. Iron is mainly stored as ferritin in the liver, spleen, and bone marrow, serving as a reservoir that releases iron when needed [7]. Serum ferritin levels correlate with total body iron stores and serve as a critical indicator of iron status. In the bloodstream, transferrin, a glycoprotein, transports most iron to cells by binding and delivering it through specific transferrin receptors [8]. Total iron-binding capacity (TIBC) measures the blood’s ability to bind iron, reflecting available transferrin binding sites [9]. TIBC is typically elevated in iron deficiency due to an increase in unbound transferrin while unsaturated iron-binding capacity (UIBC) represents the portion of transferrin that is not bound to iron, complementing TIBC in assessing iron-binding status [9]. Transferrin saturation (TSAT), calculated as the ratio of serum iron to TIBC, indicates the percentage of transferrin binding sites occupied by iron [9].

Previous studies have explored the impact of serum ferritin levels on MASLD and liver fibrosis [10]. However, the relationship between complete serum iron status and MASLD as well as liver fibrosis remains unexplored. Furthermore, previous studies have generally analyzed complete serum iron status and MASLD diagnosed by traditional ultrasound [11]. This study intends to utilize the Controlled Attenuation Parameter (CAP) and Liver Stiffness Measurement (LSM), derived from Vibration-Controlled Transient Elastography (VCTE). CAP quantifies hepatic steatosis by measuring the degree of ultrasound attenuation caused by fat deposition in the liver, providing a reliable, non-invasive alternative to liver biopsy for assessing fatty liver disease [12]. LSM evaluates liver fibrosis by quantifying the liver’s stiffness through the velocity of shear waves, serving as a crucial tool for staging fibrosis and cirrhosis [12]. The objective of this study is to investigate the cross-sectional relationship between complete serum iron status and MASLD, as well as liver fibrosis based on the National Health and Nutrition Examination Survey (NHANES) 2017-2020 cycle.

## 2. Materials and methods

### 2.1. Study population

**Fig 1:**
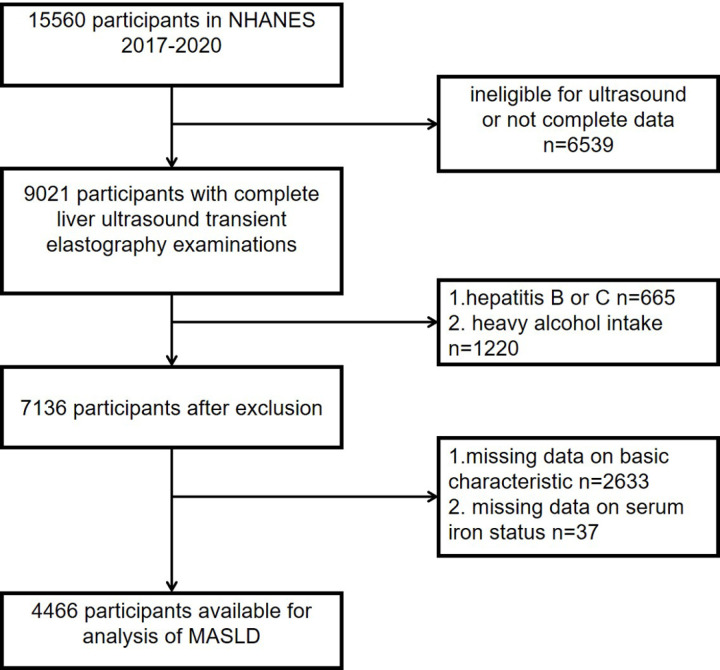
Flowchart of participant screening and selection process.

The data were sourced from the 2017–2020 NHANES cycle. NHANES is a large, population-based study that collects a wide range of health data, including clinical, demographic, and laboratory measurements. The study was supported by the National Centre for Health Statistics Research Ethics Review Board. This dataset is particularly useful for cohort studies examining the prevalence and risk factors of liver diseases such as MASLD, given its diverse participant pool and comprehensive health information. The initial cohort included 15,560 participants, with exclusions based on the following criteria: (1) incomplete liver elastography measurements (n = 6,539), (2) hepatitis B or C diagnosis (n = 665), (3) heavy alcohol consumption, defined as ≥ 3 drinks/day for males and ≥ 2 drinks/day for females (n = 1,220), (4) incomplete basic characteristic data (n = 2,633), and (5) missing serum iron status data (n = 37). Following these exclusions, the final sample size was 4,466 participants. Refer to Fig 1 for a detailed overview.

### 2.2. Measurement of serum iron status

Ferritin levels are measured using an immunoassay method that forms an antibody-antigen complex, with chemiluminescence detection used to indicate these levels. Iron concentration is quantified through a colorimetric reaction, where iron in the sample reacts with a reagent to form a colored compound. The color intensity correlates with the iron concentration. UIBC is measured using an iron-binding process in an alkaline environment to assess the unbound capacity of transferrin, with color changes indicating binding capacity; greater color intensity signifies lower binding capacity. TIBC is calculated by summing serum iron and UIBC values. TSAT is expressed as a percentage by dividing serum iron by TIBC. Detail regarding the lower limit of detection (LLOD) of different serum iron status levels and the proportion above the LLOD is provided in S1 Table. For ease of analysis, participants were stratified into four cohorts based on the quartile concentrations of complete serum iron status.

### 2.3. The definition of MASLD

The diagnosis of MASLD is based on hepatic steatosis in the absence of significant alcohol consumption or viral hepatitis. Inclusion criteria should include at least one of the following: (1) Body mass index (BMI) ≥  25 kg/m², or waist circumference (WC) ≥  94 cm for males and ≥  80 cm for females; (2) Fasting plasma glucose (FPG) ≥  5.6 mmol/L, HbA1c ≥  5.7%, a history of T2DM, or currently receiving treatment for T2DM; (3) Blood pressure ≥  130/85 mmHg or currently undergoing antihypertensive treatment; (4) Triglyceride (TG) levels ≥  1.70 mmol/L or currently receiving lipid-lowering treatment; (5) Male high-density lipoprotein cholesterol (HDL) <  1.0 mmol/L or female <  1.3 mmol/L, or currently receiving lipid-lowering treatment [5].

### 2.4. The assessment of liver steatosis and fibrosis

This study utilized VCTE to quantify hepatic steatosis and liver fibrosis. Participants were required to fast for at least 3 hours before undergoing more than 10 liver measurements, with the goal of keeping the interquartile range to median ratio below 30% to ensure measurement accuracy. Hepatic steatosis was diagnosed based on a CAP score of 238 dB/m or higher [13,14], while liver fibrosis was determined by an LSM score of 7 kPa or higher, consistent with established literature [15,16].

### 2.5. Covariate evaluation

Demographic data were collected using a standardized, self-administered questionnaire, including information on age, gender, ethnicity, education level, marital status, poverty-income ratio (PIR), alcohol consumption, physical activity (PA), and medication use. PA was evaluated using the formula: PA =  MET (metabolic equivalent of task) ×  frequency per week ×  duration of each activity. A PA value of 0 indicated the absence of physical activity. Participants were categorized based on the stipulation of reaching a minimum of 600 MET-minutes per week for adults [17]. Smoking habits were evaluated by measuring cotinine levels [18,19] and economic status was classified into three groups based on the PIR level [20]. Diabetes was diagnosed based on FPG levels of 7 mmol/L or higher, HbA1c levels of 6.5% or higher, self-reported clinical diagnosis of diabetes, or the use of diabetes medication [21]. Hypertension was diagnosed if participants had blood pressure exceeding 130/80 mm Hg or were taking antihypertensive medications [22]. Participants were classified into two groups based on alcohol consumption: the abstainer group and the moderate drinking group (1–2 drinks/day for males, 1 drink/day for females) [23].

### 2.6. Statistical methodology

Continuous variables were expressed as means and standard deviations, while categorical variables were presented as percentages. Comparisons of continuous variables were performed using weighted t-tests, and comparisons of categorical factors were conducted using chi-square tests. A linear regression model was used to analyze the relationship between serum iron status and CAP and LSM. We developed three independent analytical models to explore the complex relationships between covariates and outcomes, each model with progressively detailed adjustments. The baseline model remained unchanged, while the second model introduced specific adjustments for variables such as PIR, PA, BMI, age, ethnicity, gender, education level, and marital status. The fully adjusted third model incorporated additional factors, such as smoking, alcohol consumption, diabetes prevalence, and hypertension. As mentioned earlier, we used multivariate logistic regression to analyze the association between serum iron status and liver fibrosis in MASLD patients. Subgroup analyses were performed to assess differences in outcome measures, with gender, age, BMI, diabetes, and hypertension considered as potential moderators. Restrictive cubic spline (RCS) analysis was used to explore the potential nonlinear relationship between serum iron status and liver fibrosis in MASLD patients. The relationship between serum iron status, MASLD, and liver fibrosis was further examined through propensity score matching and logistic regression analysis. All analyses were conducted using R software (version 4.1.0, Vienna, Austria), and a p-value <  0.05 was considered statistically significant.

## 3. Results

### 3.1. Baseline features

A total of 4,466 participants were included in the main analysis. Detailed baseline characteristics of these participants are provided in S2 Table. Statistically significant differences were observed between the MASLD group and the non-MASLD group in terms of gender, smoking habits, physical activity, diabetes, hypertension, and BMI. Additionally, patients with MASLD had higher levels of age, WC, TC, TG, HbA1c, FPG, ferritin, UIBC, and TIBC, while their HDL and iron levels were lower. Statistically significant differences between the liver fibrosis group and the non-liver fibrosis group were found in gender, education level, PIR, physical PA, diabetes, hypertension, and BMI. However, no statistically significant differences were observed for TC, LDL, HDL, UIBC, and TIBC between the two groups.

### 3.2. Correlation between serum iron status and CAP

The results in Table 1 shows the association between serum iron status and CAP. In Model 1, elevated levels of ferritin, UIBC, and TIBC were significantly positively correlated with CAP compared to the reference group, while TSAT was negatively correlated with CAP in Q4 group. In Model 2, the results were similar to those in Model 1, with the exception that no significant association was found between TSAT and CAP. In Model 3, the results were consistent with those in Model 2. The result of the relationship between serum iron, TSAT, and CAP is presented in S3 Table.

**Table 1:** Correlation between serum ferritin, UIBC, TIBC and CAP.

		CAP					
		model1		model2		model3	
		β, (95% CI)	P value	β, (95% CI)	P value	β, (95% CI)	P value
Ferritin	Q1	ref	ref	ref	ref	ref	ref
	Q2	14.729(7.417,22.040)	<0.001	8.907(2.737,15.076)	0.005	8.409(2.489,14.329)	0.005
	Q3	21.986(14.544,29.428)	<0.001	10.773(4.379,17.166)	<0.001	10.205(3.975,16.435)	<0.001
	Q4	36.618(29.615,43.620)	<0.001	18.860(12.123,25.597)	<0.001	18.049(11.661,24.437)	<0.001
UIBC	Q1	ref	ref	ref	ref	ref	ref
	Q2	7.103(-0.208,14.415)	0.057	3.588(-2.484,9.660)	0.247	1.859(-3.860,7.578)	0.524
	Q3	12.252(4.796,19.709)	<0.001	7.849(1.631,14.066)	0.013	4.932(-1.039,10.904)	0.105
	Q4	14.143(6.680,21.605)	<0.001	10.792(4.024,17.561)	0.002	5.866(0.779,12.512)	0.024
TIBC	Q1	ref	ref	ref	ref	ref	ref
	Q2	7.422(-0.140,14.983)	0.054	5.322(-1.000,11.643)	0.099	-2.294(-7.332,2.745)	0.372
	Q3	12.190(4.671,19.709)	<0.001	9.987(3.801,16.173)	<0.001	0.668(-4.847,6.182)	0.812
	Q4	10.533(2.948,18.119)	<0.001	10.209(3.640,16.779)	<0.001	0.483(0.032,0.934)	0.036

### 3.3. Correlation between serum iron status and LSM

Table 2 presents the relationship between serum iron status levels and LSM. In Model 1, compared to the reference group, serum ferritin levels were significantly positively correlated with LSM (P <  0.05), while TSAT showed a negative correlation with LSM in Q4 group. In Models 2 and 3, a significant positive correlation was observed only between the highest serum ferritin levels and LSM. The result of the relationship between serum iron, TSAT, and LSM is presented in S4 Table.

**Table 2:** Correlation between serum ferritin, UIBC, TIBC and LSM.

		LSM					
		model1		model2		model3	
		β, (95% CI)	P value	β, (95% CI)	P value	β, (95% CI)	P value
Ferritin	Q1	ref	ref	ref	ref	ref	ref
	Q2	0.342(-0.051,0.736)	0.088	0.107(-0.281,0.496)	0.588	0.113(-0.287,0.513)	0.579
	Q3	0.756(0.313,1.198)	0.001	0.317(-0.152,0.787)	0.185	0.322(-0.151,0.796)	0.812
	Q4	1.017(0.604,1.429)	<0.001	0.544(0.080,1.008)	0.022	0.535(0.071,0.999)	0.024
UIBC	Q1	ref	ref	ref	ref	ref	ref
	Q2	0.059(-0.379,0.497)	0.791	0.015(-0.457,0.428)	0.948	0.074(-0.518,0.369)	0.742
	Q3	0.254(-0.209,0.717)	0.282	0.232(-0.212,0.677)	0.305	0.134(-0.307,0.574)	0.552
	Q4	2.150(-3.774,8.074)	0.477	0.368(-0.098,0.834)	0.122	0.356(-0.081,0.793)	0.110
TIBC	Q1	ref	ref	ref	ref	ref	ref
	Q2	0.096(-0.489,0.298)	0.634	0.089(-0.479,0.301)	0.655	0.126(-0.506,0.254)	0.516
	Q3	0.205(0.339,0.748)	0.460	0.277(-0.259,0.813)	0.311	0.210(-0.340,0.759)	0.454
	Q4	0.224(-0.231,0.680)	0.334	0.373(-0.088,0.834)	0.113	0.234(-0.209,0.676)	0.300

### 3.4. Correlation between serum iron status and MASLD

**Fig 2:**
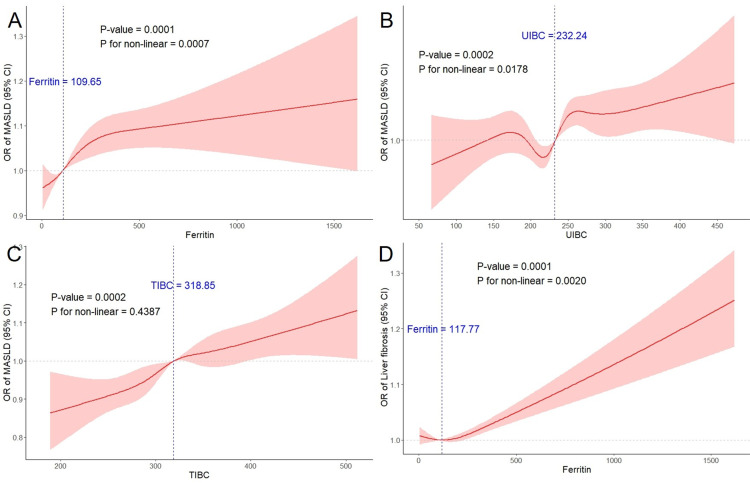
RCS analysis of the association between MASLD and (A) Ferritin, (B) UIBC, (C) TIBC, as well as Liver Fibrosis and (D) Ferritin.

Table 3 summarizes the associations between serum iron status and MASLD. Model 1 showed a significant positive association between higher levels of serum ferritin, UIBC, TIBC, and MASLD. The results of Model 2 and Model 3 were similar to those of Model 1. The logistic regression analysis examining the relationship between serum iron, TSAT, and MASLD is presented in S5 Table. A significant positive correlation between the highest serum iron levels, TSAT, and MASLD was observed in Model 1. In the RCS analysis based on Model 3, nonlinear relationship between serum ferritin, UIBC, and MASLD was observed, with the exception of TIBC (see Fig 2A-C).

**Table 3:** Correlation between serum ferritin, UIBC, TIBC and MASLD.

		MASLD						
		Q1	Q2		Q3		Q4	
			OR (95%CI)	P value	OR (95%CI)	P value	OR (95%CI)	P value
Ferritin	model1	ref	1.242(0.971-1.589)	0.085	1.776(1.368-2.306)	<0.001	2.961(2.278-3.850)	<0.001
	model2	ref	1.092(0.818-1.457)	0.550	1.429(1.056-1.935)	0.021	2.118(1.524-2.944)	<0.001
	model3	ref	1.062(0.793-1.422)	0.231	1.425(1.041-1.949)	0.027	2.126(1.508-2.996)	<0.001
UIBC	model1	ref	1.327(1.020-1.726)	0.035	1.531(1.174-1.996)	0.002	1.687(1.297-2.195)	<0.001
	model2	ref	1.250(0.927-1.685)	0.143	1.422(1.028-1.968)	0.034	1.666(1.216-2.282)	0.001
	model3	ref	1.207(0.892-1.634)	0.222	1.314(0.942-1.833)	0.107	1.437(1.041-1.984)	0.027
TIBC	model1	ref	1.472(1.134-1.911)	0.004	1.538(1.180-2.005)	0.001	1.608(1.232-2.097)	<0.001
	model2	ref	1.515(1.119-2.050)	0.007	1.593(1.174-2.162)	0.003	1.820(1.325-2.501)	<0.001
	model3	ref	1.266(0.869-1.844)	0.218	1.462(1.078-1.984)	0.015	1.627(1.175-2.252)	0.003

### 3.5. Correlation between serum iron status and liver fibrosis

Table 4 presents the associations between serum iron status and liver fibrosis. Model 1 showed a positive correlation between serum ferritin levels and the degree of liver fibrosis (P <  0.05). In Models 2 and 3, the highest serum ferritin levels were significantly positive with liver fibrosis. The logistic regression analysis assessing the relationship between serum iron, TSAT, and liver fibrosis is shown in S6 Table. In addition, in the RCS analysis based on Model 3, a significant nonlinear relationship between serum ferritin and liver fibrosis was observed (see Fig 2D).

**Table 4:** Correlation between serum ferritin, UIBC, TIBC and liver fibrosis.

		Liver fibrosis						
		Q1	Q2		Q3		Q4	
			OR (95%CI)	P value	OR (95%CI)	P value	OR (95%CI)	P value
Ferritin	model1	ref	1.409(0.960-2.067)	0.080	1.717(1.186-2.485)	0.004	2.150(1.515-3.052)	<0.001
	model2	ref	1.260(0.837-1.895)	0.268	1.403(0.942-2.089)	0.096	1.618(1.088-2.407)	0.017
	model3	ref	1.280(0.842-1.947)	0.248	1.401(0.941-2.085)	0.097	1.616(1.092-2.391)	0.016
UIBC	model1	ref	0.952(0.654-1.386)	0.797	1.213(0.853-1.725)	0.283	1.225(0.861-1.744)	0.260
	model2	ref	0.859(0.581-1.269)	0.444	1.145(0.802-1.637)	0.456	1.137(0.794-1.628)	0.483
	model3	ref	0.781(0.532-1.145)	0.205	1.014(0.709-1.450)	0.941	0.949(0.660-1.364)	0.777
TIBC	model1	ref	1.178(0.824-1.684)	0.368	1.225(0.858-1.748)	0.263	1.330(0.934-1.895)	0.114
	model2	ref	1.174(0.815-1.692)	0.389	1.270(0.876-1.840)	0.207	1.406(0.980-2.019)	0.065
	model3	ref	1.143(0.796-1.642)	0.470	1.183(0.808-1.730)	0.388	1.234(0.862-1.768)	0.251

### 3.6. Subgroup analysis

**Fig 3:**
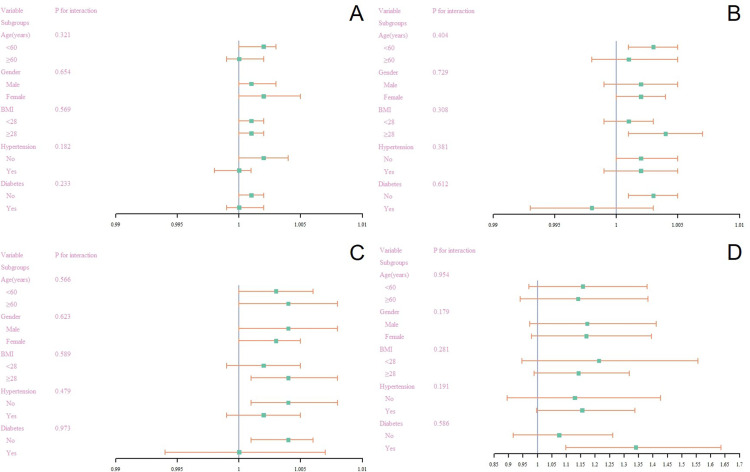
Subgroup analysis of the association between MASLD with (A) ferritin, (B) UIBC, (C) TIBC and liver fibrosis with (D) ferritin by stratified groups.

We conducted stratified multivariable regression analysis, as shown in Fig 3A-D. No significant associations were found in any subgroup analysis of serum ferritin, UIBC, TIBC, and MASLD, or the subgroup analysis of serum ferritin and liver fibrosis (all p >  0.05). The relationship between serum ferritin, UIBC, TIBC, and MASLD, as well as between serum ferritin and liver fibrosis, was further explored through propensity score matching and logistic regression analysis. The results (see S7,S8,S9 Tables) further support the robustness of our findings.

## 4. Discussion

Previous study has primarily focused on exploring the relationship between complete serum iron status and MASLD [11]. However, epidemiological data on the potential association between serum iron status and MASLD as well as liver fibrosis detected by VCTE are currently lacking. Additionally, studies using VCTE to assess the correlation between serum iron status and MASLD or liver fibrosis have mainly concentrated on ferritin [10]. To fill this gap, we analyzed the complete serum iron status and VCTE data from the NHANES study. Our findings revealed a significant positive correlation between serum ferritin, TIBC, UIBC, and MASLD, with a similar positive correlation observed between serum ferritin and liver fibrosis. These findings are consistent with previous research and remain robust after adjusting for various potential confounders [10,11]. However, our subgroup analyses did not reveal any significant differences in variables.

Hepcidin, a central regulator of iron metabolism, plays a pivotal role in maintaining iron homeostasis by inhibiting intestinal iron absorption and promoting iron sequestration in macrophages [24]. Elevated hepcidin levels, often triggered by systemic inflammation or iron overload, inhibit ferroportin, reducing iron export from hepatocytes and macrophages. This iron retention exacerbates oxidative stress and lipid peroxidation, which promote hepatocyte injury, steatosis, and fibrosis [25]. Furthermore, chronic inflammation associated with MASLD can amplify hepcidin expression, creating a feedback loop that worsens liver damage [25]. Iron overload is known to significantly affect liver function, contributing to hepatic steatosis and liver fibrosis. The pathophysiology of these conditions involves several mechanisms driven by excess iron accumulation in hepatocytes and hepatic macrophages [26]. Excess iron directly promotes oxidative stress by catalyzing reactive oxygen species (ROS) formation through the Fenton reaction [27]. ROS production leads to lipid peroxidation, inflammation, and hepatocyte damage, all of which contribute to the development of MASLD [27]. Moreover, iron overload activates hepatic stellate cells (HSCs), the primary cells responsible for collagen production during liver fibrosis [28]. Iron triggers HSC activation through oxidative stress, inflammatory cytokines, and growth factors like transforming growth factor-beta (TGF-β), which promotes fibrosis progression. Iron overload’s pro-inflammatory effects also recruit inflammatory cells, such as neutrophils and macrophages, worsening the inflammatory response [28]. Additionally, iron-induced dysregulation of lipid metabolism in hepatocytes is a key factor in MASLD pathogenesis, leading to an imbalance between lipid synthesis and breakdown [29]. Iron interferes with autophagy in hepatocytes, further impairing cellular homeostasis and exacerbating lipid accumulation [30]. The combination of oxidative stress, inflammation, fibrosis, and disrupted lipid metabolism drives progression from simple steatosis to more severe liver damage, including MASLD and liver fibrosis [29]. The dual visualization of ferritin’s association with MASLD and liver fibrosis highlights their distinct pathological mechanisms. Ferritin likely contributes to MASLD via oxidative stress and lipid metabolism dysregulation, while its impact on liver fibrosis involves hepatic stellate cell activation and collagen deposition. This approach clarifies the unique pathways ferritin influences and aligns with the study’s objective of exploring nuanced serum iron marker-liver pathology relationships.

Few studies have examined the correlation between various components of serum iron metabolism and MASLD. Earlier research has identified a significant positive association between TIBC/UIBC levels and the incidence of NAFLD, consistent with our findings [31]. Conversely, studies on iron metabolism and NAFLD or MAFLD have demonstrated a significant negative correlation between serum iron concentration, TSAT, and NAFLD/MAFLD as well as liver fibrosis [32,33]. In contrast, our study did not detect any association between serum iron concentration, TSAT, and MASLD or liver fibrosis. This discrepancy may arise because the definition of MASLD differs from that of NAFLD/MAFLD, and the criteria for diagnosing steatosis and fibrosis vary. Subgroup analysis did not identify a significant correlation between serum iron status and MASLD or liver fibrosis when considering the effect of age, consistent with previous research [10,11]. Moreover, previous studies have consistently demonstrated a stronger positive correlation between serum ferritin levels and CAP in males [10]. However, this study found no positive correlation between serum ferritin levels and MASLD after adjusting for gender, possibly because the diagnostic criteria for MASLD must not only satisfy CAP requirements but also take other metabolic impairment factors into account. In the gender subgroup analysis, no significant difference was found in the correlation between serum iron status and MASLD, consistent with the findings of Xia et al. [11]. In addition, ferritin is not only a marker of iron stores but also an acute phase response protein, which can be elevated in states of inflammation, such as those observed in steatohepatitis [34]. This characteristic of ferritin makes its interpretation in liver disease studies more complex, as elevated ferritin could reflect both iron overload and the inflammatory process involved in MASLD and liver fibrosis [35]. Transferrin, a negative acute phase protein, decreases during inflammation and may influence iron metabolism [36]. Although we did not measure transferrin directly, we assessed TIBC, UIBC, and TSAT, which provide additional insights into iron metabolism. The lack of correlation between these markers and MASLD or liver fibrosis may be due to the distinct diagnostic criteria for MASLD. Future research examining both ferritin and transferrin could further clarify their roles in MASLD pathophysiology and the inflammatory processes involved.

To our knowledge, this is the first study to explore the relationship between serum iron status, MASLD, and liver fibrosis using VCTE. However, there are several limitations to consider. First, the cross-sectional design of the NHANES dataset inherently limits the ability to establish causal relationships. It remains unclear whether abnormal iron indices contribute to liver inflammation or if they are a consequence of it. To establish causality, longitudinal studies with repeated measurements and intervention trials addressing iron overload or inflammation are needed to better understand the temporal relationship and causal mechanisms. Second, iron metabolism may change over the long-term progression of MASLD, and we are unable to infer the impact of these dynamic changes on MASLD or liver fibrosis. Thirdly, patients with advanced liver fibrosis and/or cirrhosis may develop portal hypertension, which can influence iron stores due to blood loss and altered liver function. While this study focused on iron metabolism markers, portal hypertension could have been a confounding factor that might have impacted the results, particularly in advanced cases. In addition, we acknowledge that the absence of a specific healthy control group is a limitation of this study, as it may reduce the ability to directly compare the iron status of individuals with MASLD or liver fibrosis to those without these conditions. Moreover, the reliance on self-reported data for certain variables, including alcohol consumption and PA, introduces the possibility of recall bias, which may affect the accuracy of these variables. Residual confounding factors, such as dietary intake, genetic predisposition, and other unmeasured variables, may not be fully accounted for, potentially influencing the observed associations. Furthermore, due to the lack of medication data, we cannot assess the potential influence of medications on the iron status of participants. Additionally, we were unable to investigate specific factors that may affect iron status, such as the dietary intake of iron, hepatitis and liver injury. The mechanisms by which iron status influences MASLD and liver fibrosis warrant further investigation.

## 5. Conclusion

This comprehensive cross-sectional study, involving a large cohort, identified a significant positive correlation between elevated serum ferritin, UIBC, and TIBC levels with CAP, while higher serum ferritin was also positively correlated with LSM. Furthermore, higher serum ferritin, UIBC, and TIBC were positively associated with an increased prevalence of MASLD and a significantly higher risk of liver fibrosis associated with ferritin.

## Supporting information

S1 Table Detection frequency of iron status in NHANES.
(DOCX)

S2 Table Characteristics of participants included.
(DOCX)

S3 Table Linear regression model between serum iron, TSAT and CAP.
(DOCX)

S4 Table Linear regression model between serum iron, TSAT and LSM.
(DOCX)

S5 Table Logistic regression model between serum iron, TSAT and MASLD.
(DOCX)

S6 Table Logistic regression model between serum iron, TSAT and liver fibrosis.
(DOCX)

S7 Table Characteristics of participants based on propensity score matching.
(DOCX)

S8 Table Logistic regression analysis of between serum ferritin, UIBC, TIBC and MASLD after propensity score matching.
(DOCX)

S9 Table Logistic regression analysis of between serum ferritin and liver fibrosis after propensity score matching.
(DOCX)

S1 File The data from NHANES used in our analysis.
(XLSX)

## References

[1] Targher G, Corey KE, Byrne CD, Roden M. The complex link between NAFLD and type 2 diabetes mellitus - mechanisms and treatments. Nat Rev Gastroenterol Hepatol. 2021;18(9):599–612. doi: 10.1038/s41575-021-00448-y 33972770

[2] Gofton C, Upendran Y, Zheng M-H, George J. MAFLD: how is it different from NAFLD?. Clin Mol Hepatol. 2023;29(Suppl):S17–31. doi: 10.3350/cmh.2022.0367 36443926 PMC10029949

[3] Crane H, Gofton C, Sharma A, et al. MAFLD: an optimal framework for understanding liver cancer phenotypes. J Gastroenterol, 2023, 58(10): 947–64. 37470858 10.1007/s00535-023-02021-7 PMC10522746

[4] Eslam M, Newsome PN, Sarin SK, Anstee QM, Targher G, Romero-Gomez M, et al. A new definition for metabolic dysfunction-associated fatty liver disease: an international expert consensus statement. J Hepatol. 2020;73(1):202–9. doi: 10.1016/j.jhep.2020.03.039 32278004

[5] Rinella ME, Lazarus JV, Ratziu V, Francque SM, Sanyal AJ, Kanwal F, et al. A multisociety Delphi consensus statement on new fatty liver disease nomenclature. Ann Hepatol. 2024;29(1):101133. doi: 10.1016/j.aohep.2023.101133 37364816

[6] Targher G, Byrne CD, Tilg H. MASLD: a systemic metabolic disorder with cardiovascular and malignant complications. Gut. 2024;73(4):691–702. doi: 10.1136/gutjnl-2023-330595 38228377

[7] Ganz T. Systemic iron homeostasis. Physiol Rev. 2013;93(4):1721–41. doi: 10.1152/physrev.00008.2013 24137020

[8] Nemeth E, Tuttle MS, Powelson J, Vaughn MB, Donovan A, Ward DM, et al. Hepcidin regulates cellular iron efflux by binding to ferroportin and inducing its internalization. Science. 2004;306(5704):2090–3. doi: 10.1126/science.1104742 15514116

[9] Camaschella C. Iron-deficiency anemia. N Engl J Med. 2015;373(5):485–6. 10.1056/NEJMc1507104 26222573

[10] Li C, Qu M, Tian X, Zhuang W, Zhu M, Lv S, et al. Epidemiological and transcriptome data identify association between iron overload and metabolic dysfunction-associated steatotic liver disease and hepatic fibrosis. Nutr Res. 2024;131:121–34. doi: 10.1016/j.nutres.2024.09.011 39383734

[11] Xia T, Ni J, Ni Y, Wu X, Du K, Wan X, et al. Serum iron status is associated with all-cause mortality in metabolic dysfunction-associated steatotic liver disease: a prospective, observational study. Front Endocrinol (Lausanne). 2024;15:1454193. doi: 10.3389/fendo.2024.1454193 39464186 PMC11502310

[12] Cao Y-T, Xiang L-L, Qi F, Zhang Y-J, Chen Y, Zhou X-Q. Accuracy of controlled attenuation parameter (CAP) and liver stiffness measurement (LSM) for assessing steatosis and fibrosis in non-alcoholic fatty liver disease: A systematic review and meta-analysis. EClinicalMedicine. 2022;51:101547. doi: 10.1016/j.eclinm.2022.101547 35844772 PMC9284399

[13] Shaheen M, Schrode KM, Pan D. Sex-specific differences in the association between race/ethnicity and NAFLD among US population. Front Med (Lausanne). 2021;8:795421. 34926533 10.3389/fmed.2021.795421 PMC8674562

[14] Sourianarayanane A, McCullough AJ. Accuracy of steatosis and fibrosis NAFLD scores in relation to vibration controlled transient elastography: an NHANES analysis. Clin Res Hepatol Gastroenterol. 2022;46(7):101997. doi: 10.1016/j.clinre.2022.101997 35842111

[15] Mikolasevic I, Milic S, Orlic L, Stimac D, Franjic N, Targher G. Factors associated with significant liver steatosis and fibrosis as assessed by transient elastography in patients with one or more components of the metabolic syndrome. J Diabetes Complications. 2016;30(7):1347–53. doi: 10.1016/j.jdiacomp.2016.05.014 27324703

[16] Spaur M, Nigra AE, Sanchez TR, et al. Association of blood manganese, selenium with steatosis, fibrosis in the National Health and Nutrition Examination Survey, 2017-18. Environ Res. 2022;213:113647. 35691383 10.1016/j.envres.2022.113647 PMC10031575

[17] Piercy KL, Troiano RP, Ballard RM, Carlson SA, Fulton JE, Galuska DA, et al. The physical activity guidelines for Americans. JAMA. 2018;320(19):2020–8. doi: 10.1001/jama.2018.14854 30418471 PMC9582631

[18] Tuma PA. Dietary guidelines 2020-2025: update on academy efforts. J Acad Nutr Diet. 2019, 119(4):672–4. 30005821 10.1016/j.jand.2018.05.007

[19] Reja D, Makar M, Visaria A, Karanfilian B, Rustgi V. Blood lead level is associated with advanced liver fibrosis in patients with non-alcoholic fatty liver disease: a nationwide survey (NHANES 2011-2016). Ann Hepatol. 2020;19(4):404–10. doi: 10.1016/j.aohep.2020.03.006 32376236

[20] Ogden CL, Carroll MD, Fakhouri TH, Hales CM, Fryar CD, Li X, et al. Prevalence of obesity among youths by household income and education level of head of household - United States 2011-2014. MMWR Morb Mortal Wkly Rep. 2018;67(6):186–9. doi: 10.15585/mmwr.mm6706a3 29447142 PMC5815488

[21] American Diabetes Association. Diagnosis and classification of diabetes mellitus. Diabetes Care. 2014;37 Suppl 1:S81–90. doi: 10.2337/dc14-S081 24357215

[22] Whelton PK, Carey RM, Aronow WS, et al. 2017 ACC/AHA/AAPA/ABC/ACPM/AGS/APhA/ASH/ASPC/NMA/PCNA guideline for the prevention, detection, evaluation, and management of high blood pressure in adults: executive summary: a report of the American college of cardiology/American heart association task force on clinical practice guidelines. J Am Coll Cardiol. 2018, 71(19): 2199–269. 29146535 10.1016/j.jacc.2017.11.006

[23] Cai J, Chen D, Luo W, Xu F, Feng X, Zhang L, et al. The association between diverse serum folate with MAFLD and liver fibrosis based on NHANES 2017-2020. Front Nutr. 2024;11:1366843. doi: 10.3389/fnut.2024.1366843 38567253 PMC10986760

[24] Nemeth E, Ganz T. Hepcidin and Iron in Health and Disease. Annu Rev Med. 2023;74:261–77. doi: 10.1146/annurev-med-043021-032816 35905974 PMC9943683

[25] Yang X, Wang X, Yang Z, Lu H. Iron-Mediated regulation in adipose tissue: a comprehensive review of metabolism and physiological effects. Curr Obes Rep. 2025;14(1):4. doi: 10.1007/s13679-024-00600-0 39753935

[26] Fernández-Real JM, Manco M. Effects of iron overload on chronic metabolic diseases. Lancet Diabetes Endocrinol. 2014;2(6):513–26. doi: 10.1016/S2213-8587(13)70174-8 24731656

[27] Galaris D, Pantopoulos K. Oxidative stress and iron homeostasis: mechanistic and health aspects. Crit Rev Clin Lab Sci. 2008;45(1):1–23. 18293179 10.1080/10408360701713104

[28] Cabrera E, Crespo G, VanWagner LB. Diagnosis and management of hereditary hemochromatosis. JAMA. 2022;328(18):1862–3. 36346422 10.1001/jama.2022.17727 PMC11868844

[29] Zoller H, Tilg H. Ferritin-a promising biomarker in MASLD. Gut. 2024;73(5):720–1. doi: 10.1136/gutjnl-2023-331848 38538068

[30] Yan H-F, Zou T, Tuo Q-Z, Xu S, Li H, Belaidi AA, et al. Ferroptosis: mechanisms and links with diseases. Signal Transduct Target Ther. 2021;6(1):49. doi: 10.1038/s41392-020-00428-9 33536413 PMC7858612

[31] Tan L, Zhou Q, Liu J, Liu Z, Shi R. Association of iron status with non-alcoholic fatty liver disease and liver fibrosis in US adults: a cross-sectional study from NHANES 2017-2018. Food Funct. 2023;14(12):5653–62. doi: 10.1039/d2fo04082d 37249386

[32] Zhang X, Zuo R, Xiao S, Wang L. Association between iron metabolism and non-alcoholic fatty liver disease: results from the National Health and Nutrition Examination Survey (NHANES 2017-2018) and a controlled animal study. Nutr Metab (Lond). 2022;19(1):81. doi: 10.1186/s12986-022-00715-y 36514155 PMC9749311

[33] Yu G, Liu L, Qin T, Luo Y, Song C, Chen X, et al. Associations of serum iron status with MAFLD and liver fibrosis in the USA: a nationwide cross-section study. Biol Trace Elem Res. 2024;202(1):87–98. doi: 10.1007/s12011-023-03666-4 37079265

[34] Beaton MD, Chakrabarti S, Adams PC. Inflammation is not the cause of an elevated serum ferritin in non-alcoholic fatty liver disease. Ann Hepatol. 2014;13(3):353–6. 24756010

[35] Datz C, Müller E, Aigner E. Iron overload and non-alcoholic fatty liver disease. Minerva Endocrinol. 2017;42(2):173–83. doi: 10.23736/S0391-1977.16.02565-7 27834478

[36] Zhao J, Yi Z, Deng G, Li Y, Li J, Qin M, et al. STING modulates iron metabolism to promote liver injury and inflammation in acute immune hepatitis. Free Radic Biol Med. 2024;210:367–77. doi: 10.1016/j.freeradbiomed.2023.11.038 38052276

