# Supplementary material for: Association of serum iron status with MASLD and liver fibrosis
Source: PLoS One. 2025 Apr 1;20(4):e0319057. doi: 10.1371/journal.pone.0319057 (PMC11960921; doi:10.1371/journal.pone.0319057)
Supplement: S1 Table — (DOCX) [file pone.0319057.s001.docx]

**S1 Table:** **Detection frequency of iron status in NHANES**

| Serum iron status | LLOD | proportion above the LLOD(%) |
| --- | --- | --- |
| Ferritin(ug/L) | 0.5 | 100 |
| Iron(ug/dL) | 5 | 100 |
| UIBC(ug/dL) | 17 | 99.99 |
| TIBC(ug/dL) | Not Applicable | |
| TSAT(%) |  |  |
