# Supplementary material for: Association of serum iron status with MASLD and liver fibrosis
Source: PLoS One. 2025 Apr 1;20(4):e0319057. doi: 10.1371/journal.pone.0319057 (PMC11960921; doi:10.1371/journal.pone.0319057)
Supplement: S2 Table — (DOCX) [file pone.0319057.s002.docx]

**S2 Table:** **Characteristics of participants included**

|  | Non-MASLD(n=1616) | MASLD(n=2850) | P-value | Non-Liver fibrosis(n=3764) | Liver fibrosis(n=702) | P-value |
| --- | --- | --- | --- | --- | --- | --- |
| Age (year) | 43.13(0.96) | 51.72(0.62) | <0.001 | 47.76(0.60) | 53.76(1.43) | <0.001 |
| Gender(n,%) | | | <0.001 |  |  | 0.707 |
| Male | 694(42.95) | 1417(49.72) |  | 1721(45.72) | 390(55.56) |  |
| Female | 922(57.05) | 1433(50.28) |  | 2043(54.28) | 312(44.44) |  |
| Race(n,%) | | | 0.328 |  |  | 0.122 |
| Mexican American | 120(7.43) | 358(12.56) |  | 394(10.47) | 84(11.97) |  |
| Other Hispanic | 151(9.34) | 286(10.04) |  | 361(9.59) | 76(10.83) |  |
| Non-Hispanic white | 586(36.26) | 1117(39.19) |  | 1426(37.89) | 277(39.46) |  |
| Non-Hispanic black | 482(29.83) | 632(22.18) |  | 927(24.63) | 187(26.64) |  |
| Other | 277(17.14) | 457(16.04) |  | 656(17.43) | 78(11.11) |  |
| Education level(n,%) | | | 0.701 |  |  | <0.001 |
| Less than high school | 223(13.80) | 483(16.95) |  | 573(15.22) | 133(18.95) |  |
| High school or equivalent | 354(21.91) | 655(22.98) |  | 827(21.97) | 182(25.93) |  |
| Above high school | 1039(64.29) | 1712(60.07) |  | 2364(62.81) | 387(55.13) |  |
| Marital status (n, %) | | | <0.001 |  |  | 0.077 |
| Married/cohabitant | 903(55.88) | 1793(62.91) |  | 2284(60.68) | 412(58.69) |  |
| Widowed/divorced/separated | 331(20.48) | 653(22.91) |  | 800(21.25) | 184(26.21) |  |
| Never married | 382(23.64) | 404(14.18) |  | 680(18.07) | 106(15.10) |  |
| Poverty income ratio (n, %) | | | 0.219 |  |  | 0.018 |
| <1.30 | 433(26.79) | 704(24.70) |  | 953(25.32) | 184(26.21) |  |
| 1.30-3.50 | 600(37.13) | 1120(39.30) |  | 1423(37.81) | 297(42.31) |  |
| >3.50 | 583(36.08) | 1026(36.00) |  | 1388(36.88) | 221(31.48) |  |
| Drinking status (n, %) | | | 0.263 |  |  | 0.448 |
| Non | 570(35.27) | 1053(36.95) |  | 1359(36.11) | 264(37.61) |  |
| Low to moderate | 1046(64.73) | 1797(63.05) |  | 2405(63.89) | 438(62.39) |  |
| Smoking habits (n, %) | | | <0.001 |  |  | 0.128 |
| Never | 587(36.32) | 1114 (39.09) |  | 1443(38.34) | 258 (36.75) |  |
| Moderate | 621(38.43) | 1217(42.70) |  | 1526(40.54) | 312(44.44) |  |
| Heavy | 408(25.25) | 519(18.21) |  | 795(21.12) | 132(18.80) |  |
| Physical activity (n, %) | | | <0.001 |  |  | 0.007 |
| Never | 329(20.36) | 771(27.05) |  | 898(23.86) | 202(28.77) |  |
| Insufficient | 171(10.58) | 366(12.84) |  | 446(11.85) | 91(12.96) |  |
| Constant | 1 116(69.06) | 1713(60.11) |  | 2420(64.29) | 409(58.26) |  |
| Diabetes(n, %) | | | <0.001 |  |  | <0.001 |
| Yes | 143(8.85) | 823(28.88) |  | 667(17.72) | 299(42.59) |  |
| No | 1473(91.15) | 2027(71.12) |  | 3097(82.28) | 403(57.41) |  |
| Hypertension(n, %) | | | <0.001 |  |  | <0.001 |
| Yes | 681(42.14) | 1870(65.61) |  | 2025(53.80) | 526(74.93) |  |
| No | 935(57.86) | 980(34.39) |  | 1739(46.20) | 176(25.07) |  |
| BMI (kg/m2) | | | <0.001 |  |  | <0.001 |
| <28 | 1178(72.90) | 830(29.12) |  | 1857(49.34) | 151(21.51) |  |
| ≥28 | 438(27.10) | 2020(70.88) |  | 1907(50.66) | 551(78.49) |  |
| WC(cm) | 88.86(0.66) | 106.20(0.57) | <0.001 | 97.74(0.51) | 113.38(0.67) | <0.001 |
| TG (mmol/L) | 0.84(0.02) | 1.40(0.03) | <0.001 | 1.16(0.02) | 1.40(0.06) | <0.001 |
| TC (mmol/L) | 4.68(0.06) | 4.92(0.04) | <0.001 | 4.87(0.04) | 4.60(0.08) | 0.256 |
| LDL (mmol/L) | 2.75(0.05) | 2.96(0.04) | 0.135 | 2.92(0.03) | 2.67(0.06) | 0.647 |
| HDL (mmol/L) | 1.54(0.02) | 1.32(0.02) | <0.001 | 1.42(0.01) | 1.29(0.03) | 0.897 |
| FPG(mmol/L) | 5.57(0.03) | 6.37(0.08) | <0.001 | 5.97(0.06) | 6.76(0.16) | <0.001 |
| Hb1Ac(%) | 5.41(0.03) | 5.82(0.04) | <0.001 | 5.59(0.02) | 6.17(0.10) | <0.001 |
| Ferritin(ug/L) | 114.66(6.45) | 168.12(6.19) | <0.001 | 140.63(4.52) | 199.55(6.43) | <0.001 |
| Iron(ug/dL) | 94.01(2.96) | 90.45(1.23) | 0.011 | 91.38(1.25) | 94.19(1.91) | 0.011 |
| UIBC(ug/dL) | 223.82(3.74) | 233.44(2.27) | <0.001 | 230.40(2.04) | 226.70(2.01) | 0.668 |
| TIBC(ug/dL) | 318.05(2.79) | 323.85(1.77) | 0.008 | 321.76(1.71) | 321.48(2.77) | 0.823 |
| Transferrin Saturation(%) | 29.46(0.64) | 28.57(0.42) | 0.534 | 29.03(0.39) | 28.02(0.79) | 0.592 |
