# Supplementary material for: Association of serum iron status with MASLD and liver fibrosis
Source: PLoS One. 2025 Apr 1;20(4):e0319057. doi: 10.1371/journal.pone.0319057 (PMC11960921; doi:10.1371/journal.pone.0319057)
Supplement: S3 Table — (DOCX) [file pone.0319057.s003.docx]

**S3 Table:** **Linear regression model between serum iron, TSAT and CAP**

|  |  | CAP | | | | | |
| --- | --- | --- | --- | --- | --- | --- | --- |
|  |  | model1 | | model2 | | model3 | |
|  |  | β, (95% CI) | P value | β, (95% CI) | P value | β, (95% CI) | P value |
| Iron | Q1 | ref | ref | ref | ref | ref | ref |
|  | Q2 | 0.527(-8.599,7.545) | 0.898 | 0.997(-7.669,5.676) | 0.770 | 0.083(-6.233,6.399) | 0.980 |
|  | Q3 | 0.179(-7.545,7.902) | 0.964 | 2.454(-4.274,9.182) | 0.475 | 4.675(-1.677,11.028) | 0.149 |
|  | Q4 | 5.731(-13.367,1.906) | 0.141 | 0.488(-7.191,6.216) | 0.887 | 1.260(-5.154,7.673) | 0.700 |
| TSAT | Q1 | ref | ref | ref | ref | ref | ref |
|  | Q2 | -3.582(-11.606,4.442) | 0.382 | -5.640(-12.507,1.227) | 0.107 | -4.260(-10.820,2.300) | 0.203 |
|  | Q3 | -5.176(-12.977,2.624) | 0.193 | -3.579(-10.154,2.996) | 0.286 | -1.355(-7.619,4.908) | 0.671 |
|  | Q4 | -10.454(-17.953,-2.956) | 0.006 | -5.892(-12.586,0.803) | 0.085 | -2.684(-12.586,0.803) | 0.416 |
