# Supplementary material for: Association of serum iron status with MASLD and liver fibrosis
Source: PLoS One. 2025 Apr 1;20(4):e0319057. doi: 10.1371/journal.pone.0319057 (PMC11960921; doi:10.1371/journal.pone.0319057)
Supplement: S4 Table — (DOCX) [file pone.0319057.s004.docx]

**S4 Table:** **Linear regression model between serum iron, TSAT and LSM**

|  |  | LSM | | | | | |
| --- | --- | --- | --- | --- | --- | --- | --- |
|  |  | model1 | | model2 | | model3 | |
|  |  | β, (95% CI) | P value | β, (95% CI) | P value | β, (95% CI) | P value |
| Iron | Q1 | ref | ref | ref | ref | ref | ref |
|  | Q2 | -0.230(-0.737,0.278) | 0.375 | -0.313(-0.818,0.192) | 0.551 | -0.271(-0.761,0.219) | 0.279 |
|  | Q3 | -0.423(-0.934,0.088) | 0.105 | -0.431(-0.959,0.097) | 0.110 | -0.330(-0.836,0.177) | 0.202 |
|  | Q4 | -0.428(-0.946,0.091) | 0.106 | -0.343(-0.880,0.194) | 0.210 | -0.241(-0.776,0.294) | 0.378 |
| TSAT | Q1 | ref | ref | ref | ref | ref | ref |
|  | Q2 | -0.343(-0.837,0.151) | 0.173 | -0.457(-0.951,0.038) | 0.098 | -0.408(-0.890,0.075) | 0.098 |
|  | Q3 | -0.252(-0.854,0.351) | 0.413 | -0.323(-0.937,0.290) | 0.485 | -0.215(-0.819,0.389) | 0.485 |
|  | Q4 | -0.672(-1.134,-0.209) | 0.004 | -0.653(-1.163,0.144) | 0.062 | -0.511(-0.995,0.027) | 0.069 |
