# Supplementary material for: Association of serum iron status with MASLD and liver fibrosis
Source: PLoS One. 2025 Apr 1;20(4):e0319057. doi: 10.1371/journal.pone.0319057 (PMC11960921; doi:10.1371/journal.pone.0319057)
Supplement: S5 Table — (DOCX) [file pone.0319057.s005.docx]

**S5 Table:** **Logistic regression model between serum iron, TSAT and MASLD**

|  |  | MASLD | | | | | | |
| --- | --- | --- | --- | --- | --- | --- | --- | --- |
|  |  | Q1 | Q2 | | Q3 | | Q4 | |
|  |  |  | OR (95%CI) | P value | OR (95%CI) | P value | OR (95%CI) | P value |
| Iron | model1 | ref | 0.954(0.730-1.246) | 0.730 | 0.993(0.765-1.289) | 0.959 | 0.865(0.563-0.953) | 0.021 |
|  | model2 | ref | 0.970(0.721-1.303) | 0.838 | 1.132(0.824-1.555) | 0.443 | 1.092(0.796-1.498) | 0.586 |
|  | model3 | ref | 0.999(0.743-1.342) | 0.992 | 1.203(0.871-1.662) | 0.261 | 1.152(0.836-1.588) | 0.387 |
| TSAT | model1 | ref | 0.926(0.709-1.209) | 0.570 | 0.838(0.640-1.096) | 0.196 | 0.733(1.175-2.252) | 0.003 |
|  | model2 | ref | 0.862(0.630-1.180) | 0.355 | 0.880(0.648-1.195) | 0.412 | 0.839(0.612-1.150) | 0.275 |
|  | model3 | ref | 0.891(0.649-1.222) | 0.474 | 0.947(0.694-1.292) | 0.731 | 0.915(0.666-1.256) | 0.583 |
