# Supplementary material for: Association of serum iron status with MASLD and liver fibrosis
Source: PLoS One. 2025 Apr 1;20(4):e0319057. doi: 10.1371/journal.pone.0319057 (PMC11960921; doi:10.1371/journal.pone.0319057)
Supplement: S6 Table — (DOCX) [file pone.0319057.s006.docx]

**S6 Table:** **Logistic regression model between serum iron, TSAT and liver fibrosis**

|  |  | Liver fibrosis | | | | | | |
| --- | --- | --- | --- | --- | --- | --- | --- | --- |
|  |  | Q1 | Q2 | | Q3 | | Q4 | |
|  |  |  | OR (95%CI) | P value | OR (95%CI) | P value | OR (95%CI) | P value |
| Iron | model1 | ref | 1.058(0.752-1.487) | 0.747 | 0.781(0.551-1.107) | 0.165 | 0.883(0.619-1.259) | 0.491 |
|  | model2 | ref | 1.058(0.742-1.507) | 0.756 | 0.804(0.555-1.164) | 0.247 | 1.030(0.705-1.504) | 0.879 |
|  | model3 | ref | 1.111(0.769-1.606) | 0.574 | 0.893(0.611-1.306) | 0.560 | 1.142(0.777-1.679) | 0.498 |
| TSAT | model1 | ref | 0.914(0.646-1.293) | 0.611 | 0.823(0.579-1.172) | 0.281 | 0.742(0.520-1.059) | 0.100 |
|  | model2 | ref | 0.871(0.608-1.249) | 0.454 | 0.848(0.588-1.225) | 0.380 | 0.823(0.562-1.206) | 0.318 |
|  | model3 | ref | 0.910(0.627-1.322) | 0.622 | 0.926(0.635-1.350) | 0.689 | 0.946(0.644-1.391) | 0.778 |
