# Supplementary material for: Association of serum iron status with MASLD and liver fibrosis
Source: PLoS One. 2025 Apr 1;20(4):e0319057. doi: 10.1371/journal.pone.0319057 (PMC11960921; doi:10.1371/journal.pone.0319057)
Supplement: S7 Table — (DOCX) [file pone.0319057.s007.docx]

**S7 Table.** **Characteristics of participants based on propensity score matching**

|  |  | Non-MASLD(n=1616) | MASLD(n=1616) | P-value | Non-Liver fibrosis(n=702) | Liver fibrosis(n=702) | P-value |
| --- | --- | --- | --- | --- | --- | --- | --- |
| Age (year) | | 45.18(0.65) | 55.10(0.51) | <0.001 | 54.25(0.93) | 53.08(0.96) | 0.558 |
| Gender(n,%) | | | | <0.001 |  |  | 0.707 |
| Male | | 694(42.95) | 877(54.27) |  | 391(55.70) | 390(55.56) |  |
| Female | | 922(57.05) | 739(45.73) |  | 311(44.30) | 312(44.44) |  |
| Race(n,%) | | | | <0.001 |  |  | 0.505 |
| Mexican American | | 120(7.43) | 233(14.42) |  | 85(12.11) | 84(11.97) |  |
| Other Hispanic | | 151(9.34) | 186(11.51) |  | 63(8.97) | 76(10.83) |  |
| Non-Hispanic white | | 586(36.26) | 687(42.51) |  | 280(39.89) | 277(39.46) |  |
| Non-Hispanic black | | 482(29.83) | 351(21.72) |  | 178(25.36) | 187(26.64) |  |
| Other | | 277(17.14) | 159(9.84) |  | 96(13.68) | 78(11.11) |  |
| Education level(n,%) | | | | <0.001 |  |  | 0.869 |
| Less than high school | | 223(13.80) | 303(18.75) |  | 140(19.94) | 133(18.95) |  |
| High school or equivalent | | 354(21.91) | 393(24.32) |  | 176(25.07) | 182(25.93) |  |
| Above high school | | 1039(64.29) | 920(56.93) |  | 386(54.99) | 387(55.13) |  |
| Marital status (n, %) | | | | <0.001 |  |  | 0.976 |
| Married/cohabitant | | 903(55.88) | 1107(68.50) |  | 416(59.26) | 412(58.69) |  |
| Widowed/divorced/separated | | 331(20.48) | 391(24.20) |  | 181(25.78) | 184(26.21) |  |
| Never married | | 382(23.64) | 118(7.30) |  | 105(14.96) | 106(15.10) |  |
| Poverty income ratio (n, %) | | | | 0.270 |  |  | 0.203 |
| <1.30 | | 433(26.79) | 368(22.77) |  | 181(25.78) | 184(26.21) |  |
| 1.30-3.50 | | 600(37.13) | 644(39.85) |  | 270(38.46) | 297(42.31) |  |
| >3.50 | | 583(36.08) | 604(37.38) |  | 251(35.75) | 221(31.48) |  |
| Drinking status (n, %) | | | | 0.305 |  |  | 0.091 |
| Non | | 570(35.27) | 598(37.00) |  | 295(42.02) | 264(37.61) |  |
| Low to moderate | | 1046(64.73) | 1018(63.00) |  | 407(57.98) | 438(62.39) |  |
| Smoking habits (n, %) | | | | <0.001 |  |  | 0.128 |
| Never | | 587(36.32) | 699 (43.25) |  | 1443(38.34) | 258 (36.75) |  |
| Moderate | | 621(38.43) | 647(40.04) |  | 1526(40.54) | 312(44.44) |  |
| Heavy | | 408(25.25) | 270(16.71) |  | 795(21.12) | 132(18.80) |  |
| Physical activity (n, %) | | | | <0.001 |  |  | 0.238 |
| Never | | 329(20.36) | 500(30.94) |  | 275(39.17) | 202(28.77) |  |
| Insufficient | | 171(10.58) | 210(13.00) |  | 281(40.03) | 91(12.96) |  |
| Constant | | 1 116(69.06) | 906(56.06) |  | 146(20.80) | 409(58.26) |  |
| Diabetes(n, %) | | | | <0.001 |  |  | 0.957 |
| Yes | | 143(8.85) | 659(40.78) |  | 300(42.74) | 299(42.59) |  |
| No | | 1473(91.15) | 957(59.22) |  | 402(57.26) | 403(57.41) |  |
| Hypertension(n, %) | | | | <0.001 |  |  | 0.951 |
| Yes | | 681(42.14) | 1307(80.88) |  | 175(24.93) | 526(74.93) |  |
| No | | 935(57.86) | 309(19.12) |  | 527(75.07) | 176(25.07) |  |
| BMI (kg/m2) | | | | <0.001 |  |  | 1.000 |
| <28 | | 1178(72.90) | 319(19.74) |  | 151(21.51) | 151(21.51) |  |
| ≥28 | | 438(27.10) | 1297(80.26) |  | 551(78.49) | 551(78.49) |  |
| Ferritin(ug/L) | | 122.59(5.00) | 177.20(5.49) | <0.001 | 159.84(8.29) | 204.64(8.39) | <0.001 |
| UIBC(ug/dL) | | 228.77(2.38) | 241.36(2.09) | <0.001 | 238.25(3.13) | 236.56(4.21) | 0.143 |
| TIBC(ug/dL) | | 319.14(1.91) | 325.21(1.72) | 0.008 | 322.30(2.56) | 324.89(2.47) | 0.666 |
