# Supplementary material for: Association of serum iron status with MASLD and liver fibrosis
Source: PLoS One. 2025 Apr 1;20(4):e0319057. doi: 10.1371/journal.pone.0319057 (PMC11960921; doi:10.1371/journal.pone.0319057)
Supplement: S8 Table — (DOCX) [file pone.0319057.s008.docx]

**S8 Table.** **Logistic regression analysis of between serum ferritin, UIBC, TIBC and MASLD after propensity score matching**

|  |  | MASLD | | | | | | |
| --- | --- | --- | --- | --- | --- | --- | --- | --- |
|  |  | Q1 | Q2 | | Q3 | | Q4 | |
|  |  |  | OR (95%CI) | P value | OR (95%CI) | P value | OR (95%CI) | P value |
| Ferritin | model1 | ref | 1.360(1.020-1.812) | 0.036 | 2.177(1.611-2.943) | <0.001 | 3.912(2.902-5.274) | <0.001 |
|  | model2 | ref | 1.376(0.857-2.210) | 0.187 | 1.498(0.975-2.300) | 0.065 | 2.359(1.445-3.851) | 0.001 |
|  | model3 | ref | 1.301(0.811-2.085) | 0.275 | 1.601(1.002-2.558) | 0.049 | 2.415(1.407-4.145) | 0.001 |
| UIBC | model1 | ref | 1.671(1.225-2.279) | 0.001 | 2.013(1.480-2.737) | <0.001 | 2.117(1.555-2.883) | <0.001 |
|  | model2 | ref | 1.956(1.170-3.269) | 0.010 | 2.386(1.469-3.874) | <0.001 | 2.541(1.543-4.183) | <0.001 |
|  | model3 | ref | 1.719(0.995-2.971) | 0.052 | 1.913(1.082-3.383) | 0.026 | 2.036(1.210-3.426) | 0.007 |
| TIBC | model1 | ref | 1.472(1.134-1.911) | 0.004 | 1.538(1.180-2.005) | 0.001 | 1.608(1.232-2.097) | <0.001 |
|  | model2 | ref | 1.595(0.959-2.653) | 0.072 | 2.189(1.390-3.448) | 0.001 | 1.947(1.236-3.068) | 0.004 |
|  | model3 | ref | 1.259(0.779-2.034) | 0.347 | 1.444(0.842-2.475) | 0.182 | 1.900(1.175-3.072) | 0.009 |
