# Supplementary material for: Association of serum iron status with MASLD and liver fibrosis
Source: PLoS One. 2025 Apr 1;20(4):e0319057. doi: 10.1371/journal.pone.0319057 (PMC11960921; doi:10.1371/journal.pone.0319057)
Supplement: S9 Table — (DOCX) [file pone.0319057.s009.docx]

**S9 Table.** **Logistic regression analysis of between serum ferritin and liver fibrosis after propensity score matching**

|  |  | Liver fibrosis | | | | | | |
| --- | --- | --- | --- | --- | --- | --- | --- | --- |
|  |  | Q1 | Q2 | | Q3 | | Q4 | |
|  |  |  | OR (95%CI) | P value | OR (95%CI) | P value | OR (95%CI) | P value |
| Ferritin | model1 | ref | 1.209(0.766-1.907) | 0.415 | 1.438(0.909-2.274) | 0.120 | 1.525(1.133-2.053) | 0.005 |
|  | model2 | ref | 1.094(0.690-1.734) | 0.703 | 1.288(0.814-2.037) | 0.280 | 1.659(1.024-2.686) | 0.040 |
|  | model3 | ref | 1.066(0.674-1.686) | 0.786 | 1.266(0.799-2.004) | 0.315 | 1.650(1.019-2.673) | 0.042 |
